# Broken Rotor Bar Detection in Induction Motors through Contrast Estimation

**DOI:** 10.3390/s21227446

**Published:** 2021-11-09

**Authors:** Edna Rocio Ferrucho-Alvarez, Ana Laura Martinez-Herrera, Eduardo Cabal-Yepez, Carlos Rodriguez-Donate, Misael Lopez-Ramirez, Ruth Ivonne Mata-Chavez

**Affiliations:** Department of Multidisciplinary Studies, Engineering Division, Campus Irapuato-Salamanca, University of Guanajuato, Av. Universidad S/N, Yacatitas, Yuriria 38944, Mexico; er.ferruchoalvaez@ugto.mx (E.R.F.-A.); martinez.al@ugto.mx (A.L.M.-H.); c.rodriguezdonate@ugto.mx (C.R.-D.); lopez.misael@ugto.mx (M.L.-R.); ruth@ugto.mx (R.I.M.-C.)

**Keywords:** contrast estimation, broken rotor bars, fault diagnosis, fuzzy logic, induction motors, steady state

## Abstract

Induction motors (IM) are key components of any industrial process; hence, it is important to carry out continuous monitoring to detect incipient faults in them in order to avoid interruptions on production lines. Broken rotor bars (BRBs), which are among the most regular and most complex to detect faults, have attracted the attention of many researchers, who are searching for reliable methods to recognize this condition with high certainty. Most proposed techniques in the literature are applied during the IM startup transient, making it necessary to develop more efficient fault detection techniques able to carry out fault identification during the IM steady state. In this work, a novel methodology based on motor current signal analysis and contrast estimation is introduced for BRB detection. It is worth noting that contrast has mainly been used in image processing for analyzing texture, and, to the best of the authors’ knowledge, it has never been used for diagnosing the operative condition of an induction motor. Experimental results from applying the approach put forward validate Unser and Tamura contrast definitions as useful indicators for identifying and classifying an IM operational condition as healthy, one broken bar (1BB), or two broken bars (2BB), with high certainty during its steady state.

## 1. Introduction

Rotary machines, such as induction motors (IM), have become essential tools for industrial processes due to their low cost and ruggedness [1]. These machines undergo different types of failures associated with the rotor, the stator, or the bearings due to distinct operational circumstances. An incipient fault in an IM is usually silent, and it can generate distinct types of problems, such as interruption of a production line and damage to surrounding machinery, and, in the worst scenario, it might cause a total collapse of the system, which would provoke significant economic losses for an industry [2,3]. Hence, continuous monitoring of IM is essential for detecting incipient faults in a timely manner and keeping the industrial processes working properly [4].

The presence of broken rotor bars (BRBs) is quite a difficult condition to detect, since an IM with this problem continues working without giving any hint about failure. A BRB starts as a simple crack and evolves until the bar is completely broken [5]. At this point, the IM power consumption increases; therefore, production costs also rise [6]. Hence, BRB detection has remained a subject of interest for researchers and, consequently, a considerable number of new approaches to BRB identification have emerged in recent years. These approaches look toward reliable, automatic methods of recognizing this condition with high certainty and diminishing false alarms [7]. However, many techniques proposed in the literature are invasive, and they require the IM to stop operating, making it necessary to develop more efficient fault detection techniques. These should enable fault identification during the IM steady state, without requiring to stop it to analyze the acquired data. Diverse methodologies that employ common approaches, such as motor current signature analysis (MCSA), vibration signal inspection, and voltage or magnetic flux examination, have been proposed, and they apply distinct signal processing methods, such as wavelet transform, fast Fourier transform (FFT), and entropy analysis, among many others [2].

The analysis of electric current supply to the motor is the most widely adopted and effective technique used for fault detection, since it provides cost-effective, selective, and simple means for online monitoring of electric rotary machines [8]. Hence, many methodologies based on electric current supply analysis have reported satisfactory results for fault detection in IM [9,10,11,12]. For instance, in [13], an approach based on motor current signature analysis (MCSA) is proposed for detecting BRBs in an IM by applying independent component analysis to current signal autocorrelation in the frequency domain. In [14], an approach for BRB diagnosis utilizing the third-order energy operator demodulated current signals is introduced. The adverse influence of fundamental supply frequency leakage is reduced by applying the third-order energy operator method, allowing the enhancement of characteristic BRB frequency components. In [2], the MCSA and mathematical morphology are utilized for detecting BRBs under different mechanical load conditions. A methodology for BRB detection is introduced in [6], which uses homogeneity as an index to identify the fault severity by analyzing one phase of the startup current signal supplied to the IM. In [15], a method using electric current signal fed to an IM is implemented for detecting BRBs through two Taylor–Kalman filters, in combination with a subsampling scheme, to estimate low frequencies. The analysis of the electric current signal through the Hilbert spectrum is employed in [16] for detecting incipient BRBs, considering a rotor with different levels of damage. A method for BRB detection is proposed in [17], which considers that the rotor speed varies continuously, and the supplied electric current signal is analyzed through discrete wavelet transform. In [18], a model-based support vector classification for BRB detection in an IM under full mechanical load condition is proposed; the approach extracts the used features through spectral analysis of the steady-state stator current. BRBs are detected in [19] by applying a time domain current signal analysis that consists of an oriented gradient histogram computation, an intensity gradient and an edge direction extraction from the current signals. Other techniques using distinct signal analyses, or particular examination approaches, have also been proposed. For instance, in [20], a methodology for BRB detection in IM through spectral analysis of vibration signals is presented. In [21], cyclic modulation spectrum and fast spectral correlation are combined with the Teager–Kaiser energy operator in the frequency domain for diagnosing BRBs. The method computes the time–frequency representation of the vibration signal, utilizing the short-time Fourier transform. The Teager–Kaiser energy operator is utilized for enhancing fault features, which are further intensified by calculating the spectral coherence and the enhanced envelope spectrum. BRBs are detected in [22] through the spectral analysis of the transient stator current signal during the counter-current braking. In [23], BRBs are detected by analyzing the air-gap rotational magnetic field measured in distinct stator regions. Fundamental components of differential voltages detect variations in the magneto-motive force due to rotational magnetic field distribution under the BRB condition. In [24], a technique based on the Hilbert transform and a neural network is presented for BRB diagnosis in IM; the Hilbert transform extracts the stator current envelope, which is used as an input to the neural network for diagnosing BRBs. In [25], a method for broken bar detection is proposed based on convolutional neural networks and the time–frequency representation of the motor current signals during the IM startup transient through the short-time Fourier transform. Many of the above-mentioned techniques offer high efficiency for BRB identification; however, their computational complexity prevents them from being utilized on online applications [26]. Furthermore, most of these techniques are used during the IM startup transient, since under this regime, the BRBs are easier to detect, despite an IM usually operating under a steady-state condition. Hence, BRB detection is still an open issue for researchers searching for highly accurate techniques, as BRBs are some of the most frequent and hard to detect faults in IM. BRB detection is even more difficult to perform during the steady state and under low mechanical load [27,28].

In this investigation, a strategy based on the analysis of the steady-state electric current signal fed to an IM, by estimating its contrast, is presented as a novel, low-cost computational technique for BRB recognition on the fly. The main contribution in this paper is the use of electric current signal contrast as an indicator for determining BRB presence and fault severity in an IM, since contrast has never been used as a marker for diagnosing IM to the best of the authors’ knowledge. However, it is worth noting that contrast has been largely and mainly used in image processing for analyzing texture [29,30]. From its meaning in vision, contrast provides information about abrupt variations in color and brightness among the objects within the same field of view. For a gray-level-scale image, the contrast is at its maximum when a purely black point is clearly different from a purely white point. Hence, considering that a faulty condition can modify some features of the current signal fed to the IM, by adding some low-frequency components that change the signal waveform shape in time and frequency, contrast should be suitable for identifying these fault-related variations on the waveform of the steady-state current signal fed to an IM. These changes should help to identify and discriminate among different IM states: healthy (HLT), one broken bar (1BB), and two broken bars (2BB) if the motor electric current signal is taken as a gray-level measure. Therefore, in this work, the viability of using contrast as a pointer to indicate the presence of BRBs in IM is also examined as a second contribution by employing two distinct contrast definitions, which are compared with each other to identify the one offering the best result for BRB detection and identification. The experimentally obtained results demonstrate that the proposed methodology relying on contrast estimation is highly efficient in diagnosing and classifying BRB faults in an IM, even under a low mechanical load, matching recently proposed approaches in reviewed literature, but with a lower computational complexity, thereby making it suitable to be applied in online detection and classification of BRBs.

The remainder of the manuscript is organized as follows: Section 2 describes the theoretical background on motor current signal analysis, contrast and fuzzy logic; the experimental setup is described in Section 3; the results obtained are given and compared against those from previous approaches in Section 4; finally, Section 5 provides some conclusions.

## 2. Theoretical Background

### 2.1. Motor Current Signal Analysis

The theoretical basis of motor current signal analysis relies on the potentiality of faulty conditions to influence the magnetic flux in the motor air gap. Electric current signal analysis is the most used technique in predictive maintenance for detecting and diagnosing IM faults due to its non-invasive nature. Its essence consists of acquiring and analyzing one or multiple phases of electric current signal supplied to the IM stator [31,32].

### 2.2. Contrast

Contrast, a textural feature commonly used for image classification and initially proposed by Haralick [33], is described as the recorded, perceived or reproduced tonality difference between a pair of black and white dots. Contrast can be referred to as a measure of detail preservation. In an image, the contrast between two pixels is at its maximum when their gray levels have opposite values (0 and 255) and at its minimum when they have similar gray levels; however, it is feasible to determine contrast for 2D, and 1D signals. Haralick used gray-level co-occurrence matrices *P_i,j_*, assuming that the texture content information in an image is specified by the matrix *P_i,j_*, which indicates the occurrence of two neighboring resolution cells separated by the distance *d* on the image, one with gray tone *i* and the other with gray tone *j*. Therefore, the co-occurrence matrices are functions of the angular relationship between neighboring resolution cells as well as the distance between them [33]. For this reason, Unser [34] introduced the difference histogram as an equivalence to the gray-level co-occurrence matrix, which shows benefits in required memory storage and computing time [6].

According to Unser [34], the variation between two points from *N* samples of a digital signal *L*(*n*) is defined by

(1) $$D_{n,d}=L(n)-L(n+d)$$

where *n* ∈ {0, 1, 2, …, *N* − 1}, and it is considered that the two points are separated by a fixed position *d*, and that there are at least *N_g_* distinct gray levels in *L*(*n*). Therefore, the histogram of differences *h_d_*(*j*) is defined by

(2) $$h_{d}\left(j\right)=card\{n\in N,D_{n,d}=j\}$$

where *j* = −*N*_g_ + 1, −*N*_g_ + 2, …, *N*_g_ − 2, *N*_g_ + 1 and the total number of counts *T* is given by

(3) $$T=\sum_{j}h(j)$$

A normalized difference histogram *h*_d_(*j*) is used for computing the difference in joint-probability function *P_D_*(*j*) by

(4) $$P_{D}(j)=\frac{h_{d}(j)}{T}$$

Distinct features can be defined from difference histograms; one of them is contrast *C*, which is given by

(5) $$C=\sum_{j}j^{2}\cdot P_{D}(j)$$

On the other hand, Tamura [35] proposed an alternative contrast definition that does not change the image structure but is intended to change the quality of the image. When two patterns only vary in their corresponding gray-level assortment, the dissimilarity between their contrast can be quantified [35]. In this regard, Tamura takes into consideration alternative factors to describe contrast, namely:

- Adjustable bounds of gray levels;
- Polarization of the black and white dispersion on the gray-level histogram or the correlation between black and white regions.

The gray-level approximation distribution can be perceived in the variance *σ*^2^, and kurtosis *α*_4_ provides the polarization degree, which assesses analytically the concentration level of variable values around the midpoint (mean) *μ*_4_ of their frequency distribution, which is defined as

(6) $$\alpha_{4}=\frac{\mu_{4}}{\sigma^{4}}$$

where *α*_4_ is the fourth central moment and *σ* is the standard deviation (*σ*^2^ is the variance). Consequently, contrast is determined as follows:

(7) $$F_{cont}=\frac{\sigma }{\alpha_{4}{}^{\eta }}$$

where *η* is experimentally defined as a positive number [35].

### 2.3. Fuzzy Logic Classifier

**Figure 1:**
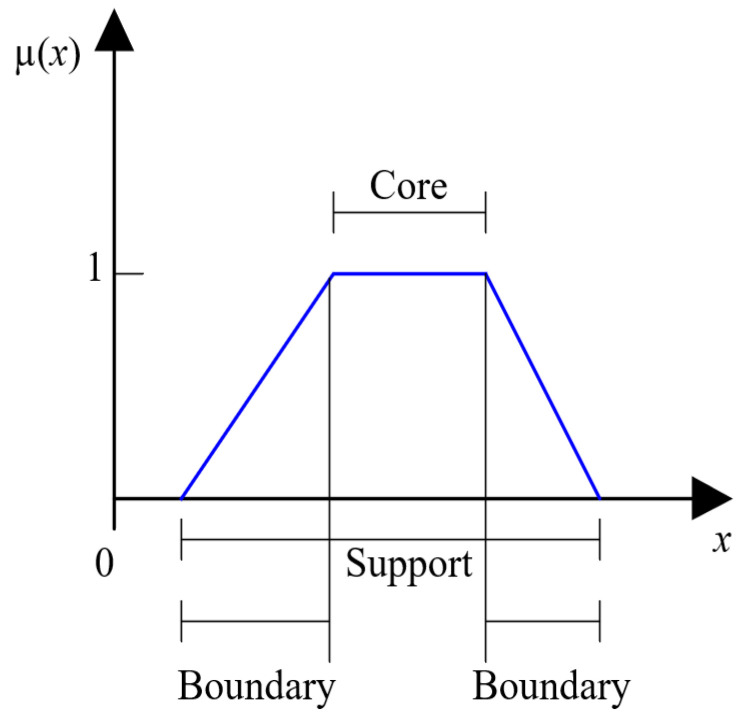
Components of a membership function.

Fuzzification is the process of making crisp quantity fuzzy. This can be done by recognizing many of the quantities considered deterministic as not deterministic, because they have considerable uncertainty. Whether the form of uncertainty arises due to imprecision, ambiguity or vagueness, the variable is probably fuzzy and can be represented by a membership function, as depicted in Figure 1, where an input value to the membership function is mapped into a quantity in the close interval [0, 1] [36,37].

- For a membership function that represents a fuzzy set *A*, the CORE is defined as the area of the universe that has the characteristic of belonging completely to set *A*. In other words, the CORE comprises all the elements *x* in the universe in which *µ_A_*(*x*) = 1.
- The SUPPORT in a membership function representing fuzzy set *A* is determined by the region of the universe characterized by non-zero values of membership elements in set *A*. This implies that the SUPPORT comprises all the elements *x* in the universe in which *µ_A_*(*x*) > 0.
- The BOUNDARY of a membership function representing fuzzy set *A* is defined as the area of the universe containing elements with non-zero membership values that do not belong completely to set *A*. This means that the BOUNDARIES of a membership function comprise those elements *x* of the universe in which 0 < *µ_A_*(*x*) < 1 [36].

Fuzzy inference uses a set of *M* if–then rules and a fuzzy rule base that contains this set. Each rule has an antecedent or premise and a consequent. The antecedent part is a Boolean expression of simple sentences on individual features *x*_1_, *x*_2_, *x*_3_, …, *x_n_*. The Mamdani–Assilian model (the logical model) is used as the fuzzy if–then system, where the input and the output are represented by Boolean expressions through linguistic terms [37].

Defuzzification is an inverse process to fuzzification, which transforms a fuzzy quantity into a specific number. In this study, the centroid method is used to carry out the defuzzification process [36,37].

## 3. Experimental Setup

**Figure 2:**
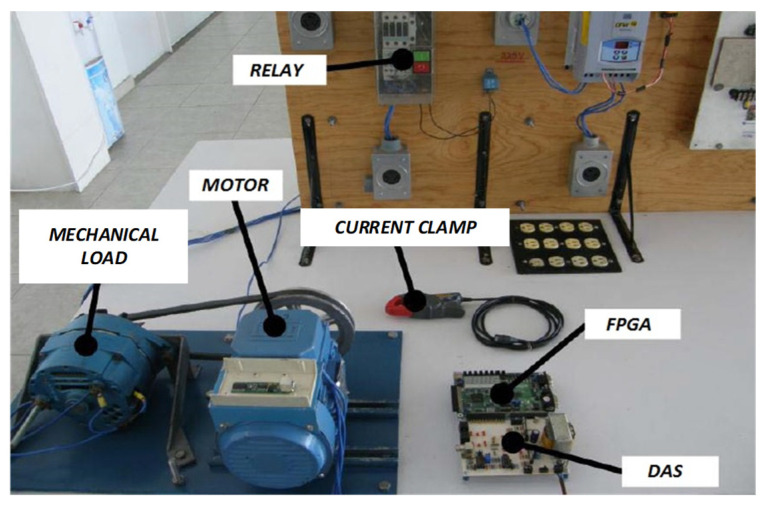
Experimental setup.

**Figure 3:**
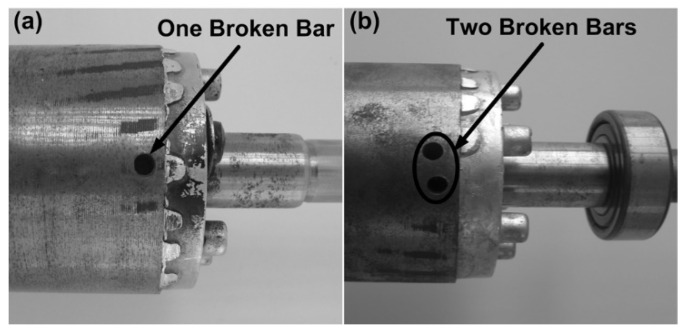
Motor conditions.

Figure 2 shows the experimental setup where a 1-HP, three-phase induction motor model WEG 00136APE48T with two poles and 28 bars is used for testing the proposed methodology. The motor is fed through a 60 Hz, 220 V power supply. The mechanical load is applied utilizing an ordinary alternator, which represents a quarter of the motor total load. The steady-state electric current signal fed to the IM is acquired utilizing a current clamp model i200 from Fluke and conditioned through a data acquisition system (DAS) based on the analog-to-digital converter ADS7809 from Texas Instrument Corporation with 16-bit resolution [38]. The frequency of acquisition $1D453_{0}$ is set to 1.5 kHz to obtain all the information that exists up to the tenth harmonic and further, since these data contain all the signal energy [6], resulting in 4096 samples being obtained. Three different IM conditions are considered in this study: an IM with one broken rotor bar (1BB), an IM with two broken rotor bars (2BB) and a healthy IM (HLT). These conditions are generated artificially by drilling holes to break one and two bars without causing any damage to the rotor shaft. Each hole has a diameter of 7.938 mm, as shown in Figure 3 for both faulty conditions, 1BB and 2BB.

**Figure 4:**
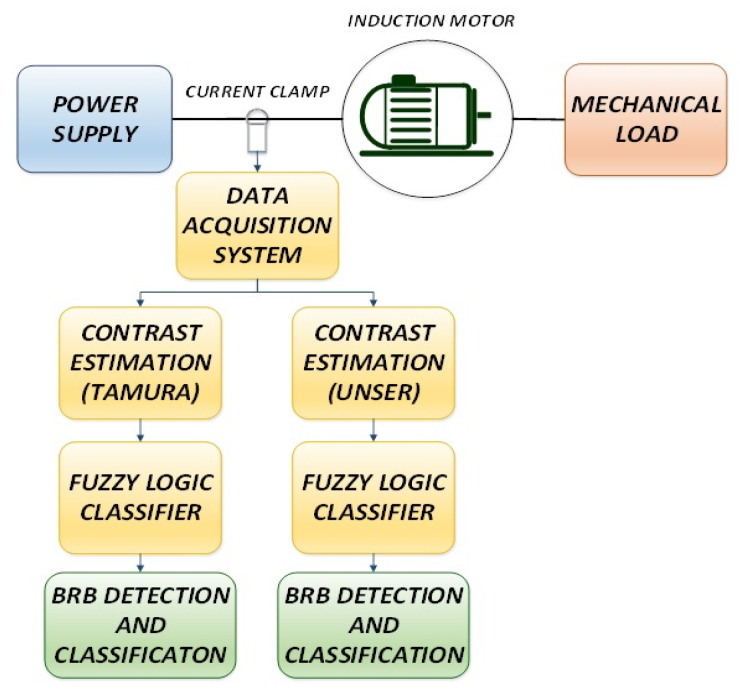
Proposed methodology for BRB detection.

The proposed methodology to detect and classify incipient BRB faults in an IM through the analysis of its steady-state electric current signal by means of contrast calculation and fuzzy-logic categorization is depicted in Figure 4.

**Figure 5:**
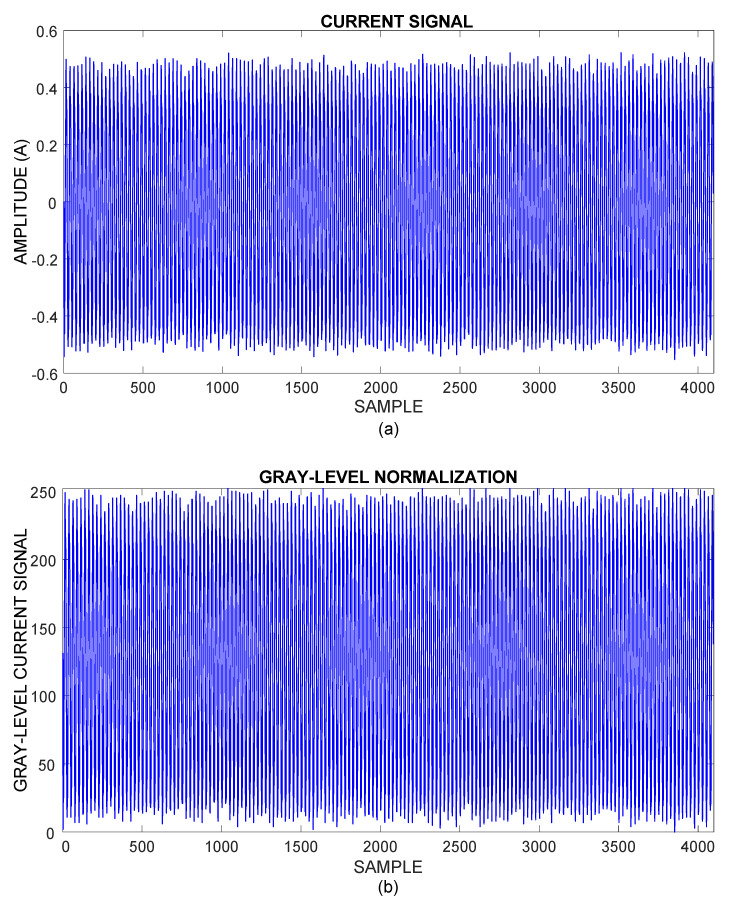
(**a**) Current signal acquired. (**b**) Gray-level current signal.

Figure 5a shows the steady-state current signal from one phase of the power supply feeding the induction motor. The acquired current data are taken into gray scale levels from 0 to 255 through a linear conversion, without modifying the electric current waveform as depicted in Figure 5b.

**Figure 6:**
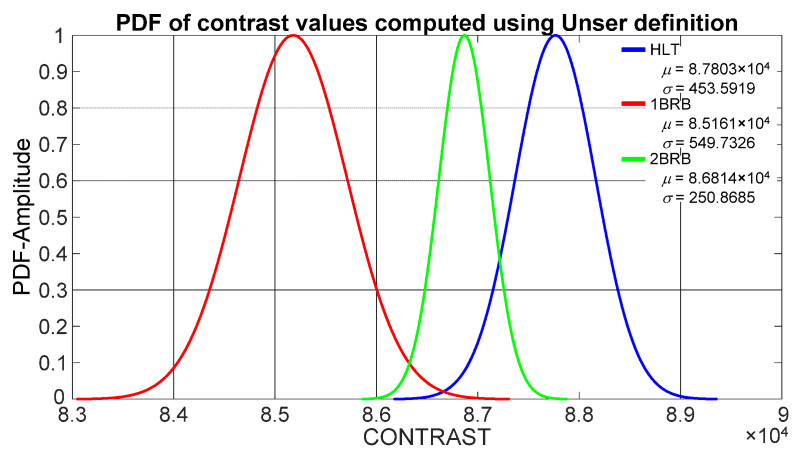
PDFs from contrast values computed through Unser definition for a healthy motor (HLT), a motor with one broken rotor bar (1BRB) and a motor with two broken rotor bars (2BRB).

Unser [34] defined contrast based on the difference histogram as a probability Function (4), where *d* is the distance separation between two pixels, and it was found heuristically that the best results are obtained when *d* = 10. Contrast values are obtained for each motor state, namely, HLT, 1BB and 2BB, at this pixel distance (*d* = 10). The mean (*μ*) and standard deviation (*σ*) of the contrast values obtained through Unser definition for a healthy motor, a motor with one broken bar and a motor with two broken rotor bars show that the corresponding probability density functions (PDFs) overlap each other, as shown in Figure 6. Hence, fuzzy logic classification is required to improve the accuracy of operational condition detection. 

**Figure 7:**
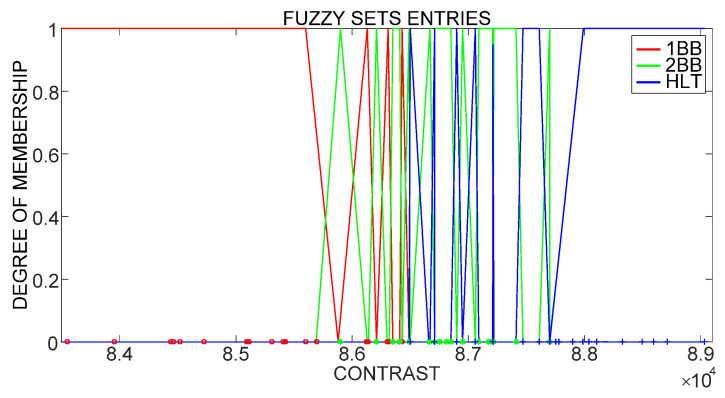
Fuzzy set entries based on Unser definition of contrast.

Fuzzy logic classification is carried out utilizing input fuzzy sets that contain the contrast computation results obtained through Unser definition for each analyzed IM condition, as shown in Figure 7. The characteristics of a fuzzy logic classifier are: Mamdani-type fuzzy inference, centroid defuzzification technique and 21 if–then rules with simple sentences of one input/one output to simplify and improve classifier performance.

**Figure 8:**
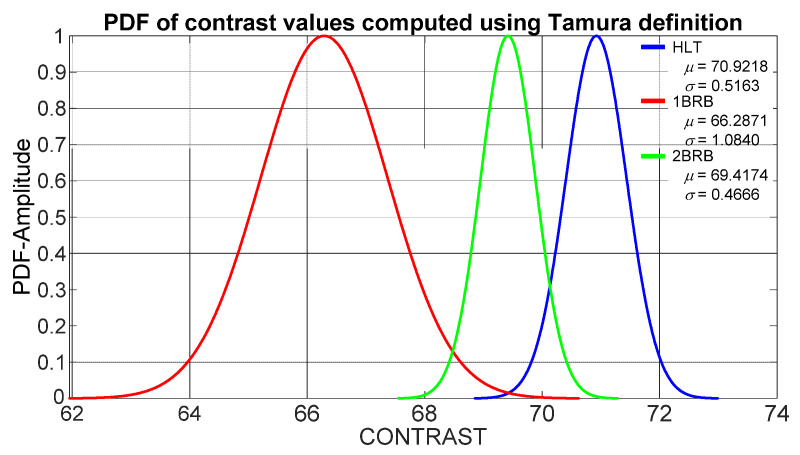
PDFs from contrast values computed through Tamura definition for a healthy motor (HLT), a motor with one broken rotor bar (1BRB) and a motor with two broken rotor bars (2BRB).

On the other hand, Tamura [35] defined kurtosis-based contrast as in (7), with *n* = 1/4. Each motor state (HTL, 1BB and 2BB) produces a contrast value. The mean (*μ*) and standard deviation (*σ*) of contrast values obtained through Tamura definition for a healthy motor, a motor with one broken bar and a motor with two broken rotor bars show that the corresponding probability density functions (PDFs) overlap each other, as shown in Figure 8. Hence, as in the Unser definition case, fuzzy logic classification is required to improve the accuracy of operational condition detection.

**Figure 9:**
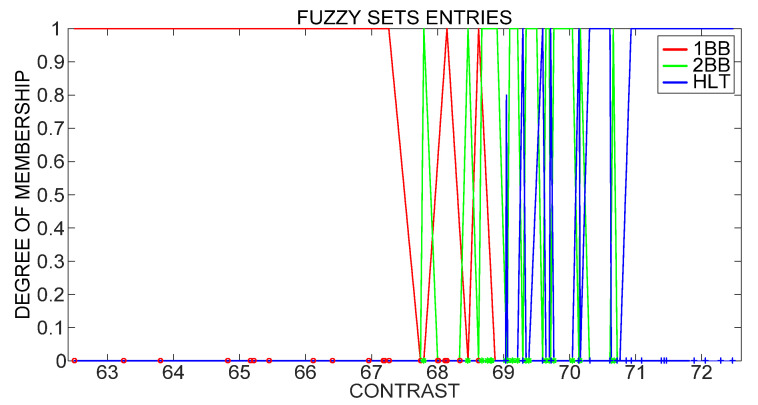
Fuzzy sets entries based on the Tamura definition of contrast.

The fuzzy logic classes are produced utilizing input fuzzy sets that contain the contrast computation results obtained from Tamura definition for each analyzed IM condition, as can be observed in Figure 9. The characteristics of a fuzzy logic classifier for this case are: Mamdani-type fuzzy inference, centroid defuzzification method and 19 if–then rules with simple sentences of one input/one output.

**Figure 10:**
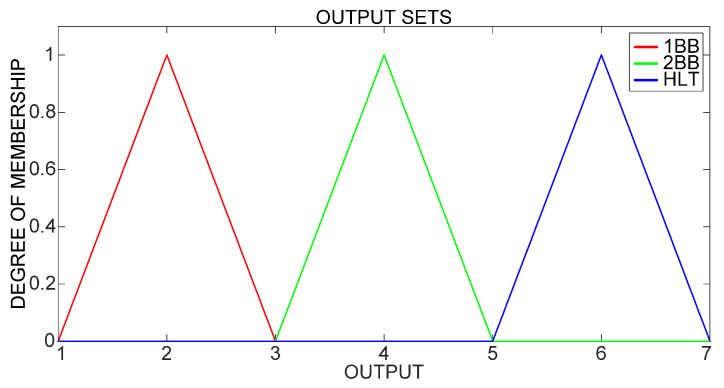
Output fuzzy sets.

The output sets of both classifiers differentiate among three IM operational conditions: HLT, 1BB and 2BB, as shown in Figure 10.

## 4. Results and Discussion

For the proposed method, a hold-out type dataset is employed [37], where 180 trials are performed on each IM condition. The first 120 experiments from each dataset are used for training the system, and the remainder are used for testing the proposed method’s effectiveness. Table 1 and Table 2 describe the proposed methodology performance in identifying and classifying BRBs, utilizing Unser and Tamura contrast definitions, respectively, through confusion matrices. Meanwhile, Table 3 shows the overall effectiveness of the proposed approach, considering each contrast definition.

From obtained results, it can be observed that even though Tamura (7) defines contrast in a simpler way than Unser (4), the proposed methodology using the latter provides higher effectiveness than using the former, reaching up to 100% accuracy in identifying and classifying BRBs. Furthermore, the computational complexity of using Unser definition is lower than using the Tamura one, since Unser description works with difference histograms instead of co-occurrence matrices. In this regard, the proposed methodology implemented in an Intel Core i7-8750H microprocessor at 2.20 GHz, utilizing MATLAB 2020a, elapses for 14.052 ms utilizing the Unser definition of contrast, whereas it lasts for 19.723 ms when applying Tamura definition.

### Discussion

A comparison of the introduced methodology against distinct techniques for broken bar detection, regarding their corresponding efficacy and signal processing complexity, is shown in Table 4. From the experimentally obtained results, the introduced technique shows that it can detect BRBs with high certainty during the steady state. Using the Unser definition of contrast exhibits higher effectiveness for BRB detection than using Tamura definition, at a slightly longer processing time. The proposed method can detect and classify the induction motor operational condition as healthy, 1BB, or 2BB, with high effectiveness of up to 98.3%, 100% and 100%, respectively, surpassing most state-of-the-art schemes in this area. Previous works in Table 4 that reach high efficacy attain it through combining three or more complex processing techniques, whereas most of them identify BRBs in a qualitative style, relying on subjective interpretation of a chart. Furthermore, most previous works in the reviewed literature carry out BRB detection during the induction motor startup transient, since the fault is easier to observe under this regime because of the increased current in the rotor circuit and sometimes under the condition of heavy load to amplify the effects of BRBs in the stator current [39]. Under the proposed approach, the identification and classification of the IM operational condition are performed with high certainty during its steady state at a low mechanical load, which means that the machine can be analyzed while it is working, without the necessity of turning it off and stopping the entire process. The experimentally obtained results demonstrate the feasibility of using contrast, which has been mostly used to analyze texture in image processing, but not as an index for IM diagnosis, as a reliable indicator for identifying and classifying BRBs in IM.

## 5. Conclusions

BRBs are among the most recurring fault conditions in IM that are quite hard to detect, increasing IM power consumption without giving any indication of failure. Therefore, BRB detection has remained a subject of interest for investigation. Hence, from the experimentally obtained results and their comparison with those from state-of-the-art schemes, the following conclusions can be inferred:

- Most approaches in the literature require the IM to stop and be put on a heavy load to be applied. It is desirable to have a reliable BRB detection technique that can be applied during the IM steady state under low load.
- In this work, a novel method for BRB detection, through analysis of the IM current signal by contrast estimation during its steady state, is proposed.
- Unser and Tamura definitions of contrast have been widely used in image processing for the analysis of texture; however, to the best of the authors’ knowledge, this index has never been used for detecting faults in IM.
- Experimentally obtained results validate that the technique put forward is able to detect and classify the induction motor operational condition as healthy, 1BB, or 2BB, with high effectiveness.
- The introduced method surpasses other approaches in state-of-the-art schemes in this area, which usually perform BRB detection by relying on subjective interpretation of a chart.
- The Unser definition of contrast provides higher effectiveness for BRB detection and classification than that of Tamura, with lower computational complexity and processing time.
- Contrast estimation from one phase of the electric current power supply is asserted as a useful indicator to identify and classify BRBs in an IM, even under low mechanical load.

Future work will focus on assessing contrast suitability for detecting other electrical and mechanical faults in IM. It will also consider combining other signal processing techniques to retrieve distinct signal features as well as evaluating different types of classifiers to improve fault detection and classification accuracy.

## Figures and Tables

**Table 1:** Confusion matrix using Unser contrast definition for BRB detection and classification.

IM Condition	HLT	1BB	2BB
**HLT**	59	0	1
**1BB**	0	60	0
**2BB**	0	0	60

**Table 2:** Confusion matrix using Tamura contrast definition for BRB detection and classification.

IM Condition	HLT	1BB	2BB
**HLT**	57	0	3
**1BB**	0	60	0
**2BB**	0	0	60

**Table 3:** Effectiveness results for identifying and classifying BRBs, utilizing contrast as a detection index.

Contrast Definition	IM Condition		
	HLT	1BB	2BB
**Unser**	98.3%	100%	100%
**Tamura**	95%	100%	100%

**Table 4:** Proposed methodology performance comparison.

Reference	Method	Detected Fault	Analyzed Signal	Motor State	Accuracy Rate
Aydin et al. [40]	1. Preprocessing signal Hilbert transform 2. Boundary analysis 3. Fuzzy decision tree (FDT)	1BB 2BB	Current signal	Not reported	98.75%
Fernandez-Cavero et al. [41]	1. Dragon transform	1BB	Current signal	Startup transient	Qualitative
Haiyang Li et al. [42]	1. Bandpass filter 2. Normalized frequency domain energy operator 3. Spectral analysis	1BB 2BB	Current signal	Steady state	Qualitative
Younes Soleimani et al. [23]	1. Air-gap rotational magnetic field analysis	1BB 2BB 3BB	Induced voltage in dual search coils	Not reported	Qualitative
Weiguo Zhao et al. [43]	1. Multivariate relevance vector machine with multiple Gaussian kernels 2. Principal components analysis 3. Bacterial foraging algorithm 4. Levy flight	1BB 2BB 3BB	Current signal	Not reported	80-95%
Mina Abd-el-Malek et al. [44]	1. Hilbert transform 2. Statistical analysis	Half broken bar 1BB 1.5BB	Current signal	Startup transient	Qualitative
Rangel-Magdaleno et al. [16]	1. Hilbert transform 2. Statistical analysis	Half broken bar 1BB 1.5BB	Current signal	Startup transient	99%
**Proposed methodology**	**1. Contrast computation** **2. Fuzzy logic**	**1BB** **2BB**	**Current signal**	**Steady state**	**98.3%**

## Data Availability

Not applicable.
